# The hospitalization burden of inflammatory bowel disease in a southwestern highland region of China: a territory-wide study from 2015 to 2020

**DOI:** 10.3389/fmed.2024.1410714

**Published:** 2024-06-07

**Authors:** Yan Tao, Maojuan Li, Huabin Gao, Yang Sun, Fengrui Zhang, Jing Wu, Hao Liang, Liping He, Min Gong, Junkun Niu, Yinglei Miao

**Affiliations:** ^1^Department of Gastroenterology, The First Affiliated Hospital of Kunming Medical University, Kunming, Yunnan, China; ^2^Yunnan Province Clinical Research Center for Digestive Diseases, Kunming, Yunnan, China; ^3^Yunnan Evaluation Center for Medical Service and Administration, Kunming, Yunnan, China;; ^4^School of Public Health, Kunming Medical University, Kunming, Yunnan, China

**Keywords:** inflammatory bowel disease, comorbidities, hospitalization costs, ulcerative colitis, Crohn’s disease

## Abstract

**Background:**

Yunnan, a southwest highland and newly industrialized region of China, has an unknown hospitalization burden of inflammatory bowel disease (IBD). The study was conducted to explore territorial hospitalization burden of IBD.

**Methods:**

The formatted medical records of patients with IBD were collected from a territory-wide database in Yunnan Province, China, from 2015 to 2020. General characteristics of the study population were reported using descriptive statistics. To evaluate the length of stay, hospitalization costs, surgery, complications, and trends in patients with inflammatory bowel disease. The logistic regression analysis was established to explore the factors affecting the hospitalization costs.

**Results:**

A total of 12,174 records from 8192 patients were included. The annual hospitalization cost of IBD in Yunnan Province increased significantly from 2015 to 2020. From 2015 to 2020, the regional hospitalization burden of IBD increased, but it represented a decline in cost per hospitalization (*r* = −0.024, *P* = 0.008) and the length of stay (*r* = −0.098, *P* < 0.001). Surgery rates for hospitalized patients with Crohn’s disease (CD) did not decrease (*r* = −0.002, *P* = 0.932), and even increased for patients with ulcerative colitis (UC) (*r* = 0.03, *P* = 0.002). The costs per hospitalization were $ 827.49 (540.11–1295.50) for UC and $ 1057.03 (644.26–1888.78) for CD. Among the identifiable cost items during the period, drug costs accounted for the highest proportion, accounting for 33% and 37.30% in patients with UC and CD, respectively. Surgical intervention [OR 4.87 (3.75–6.31), *P* < 0.001], comorbidities [OR 1.72 (1.52–1.94), *P* < 0.001], complications [OR 1.53 (1.32–1.78), *P* < 0.001], and endoscopy [OR 2.06 (1.86–2.28), *P* < 0.001] were predictor of high hospitalization costs.

**Conclusion:**

The increasing burden of IBD is noteworthy a newly industrialized region of China. Interventions targeting surgery, complications, and comorbidities may be effective means of controlling the increasing hospitalization costs of IBD in the regions.

## Introduction

Inflammatory bowel disease (IBD), including Crohn’s disease (CD) and ulcerative colitis (UC), is a chronic, progressive, and incurable immune-mediated gastrointestinal disorder with complications and extraintestinal manifestations (1–3). Patients with IBD are hospitalized for dilemmas such as recurrence, surgical intervention, complications, or diagnosis. In western countries, the annual aggregate economic burden of CD was three times higher than for the general population (4). Biologics are the fastest-growing segment of the prescription drug market, drawing on Western experience. The cost burden is expected to worsen as treatment modalities develop and biologics are introduced earlier (5). Curative medicine and advances in diagnostic technology despite allow earlier diagnosis and longer survival for patients with IBD. As the patients into adulthood and old age, concurrent comorbidities and treatment-related adverse events present management challenges (6, 7). Understanding geographic differences is important to formulate effective strategies for the prevention and treatment of IBD (8).

Global data revealed that the incidence of IBD has stabilized in Western countries, but the overall prevalence is still increasing due to improved survival and previous high incidence (3). In some newly industrialized countries, the incidence and prevalence of IBD is increasing and have not yet peaked (9, 10). To put this into context, patients with IBD will steadily increase in the near future. With the emergence of IBD in newly industrialized regions, it is important to analyze the manifestations and burden of these populations to coordinate the appropriate management (11).

Consistent with the global trend, patients with IBD are on the rise in China (6, 12). The few available data on the burden of IBD in China were mainly concentrated in developed regions (13, 14). Yunnan Province (47.22 million people in 2020), a southwest highland and newly industrialized region of China, may be mimicking the early epidemiological stages of IBD in the west. Studies prior to 2014 have reveal that the incidence and prevalence of IBD in the provincial capital (Kunming) were lower than those in western countries and developed areas of China (9, 12), but the incidence and prevalence of IBD were increasing (15, 16). Since then, multiple interventions had been used to combat the issue, but the burden of IBD in this region is a mystery. A territory-wide database was used to assess the hospitalization burden of IBD in Yunnan Province. Data in this region will help to predict the burden of IBD in less developed region worldwide.

## Methods

### Data source

The data were derived from a database of the hospital quality management and performance evaluation platform, which is affiliated to Health Commission of Yunnan Province, China. The database aggregates more than 90 percent of the regional hospitalization records and is a mandatory regional database for hospital certification. The database includes information on demographics, residence, length of stay, diagnosis, surgery and costs. The records were completed by the attending physician, and the diagnosis were then coded by a certified professional medical coder at each hospital according to the International Statistical Classification of Diseases and Related Health Problems coding system. The front sheet of standardized electronic medical records was submitted to the database in an automatic filing manner. Nearly 40 million records were recorded in the database between 2015 and 2020. As the records were used for hospital performance evaluation, the data were highly accurate.

### Data collection

**FIGURE 1:**
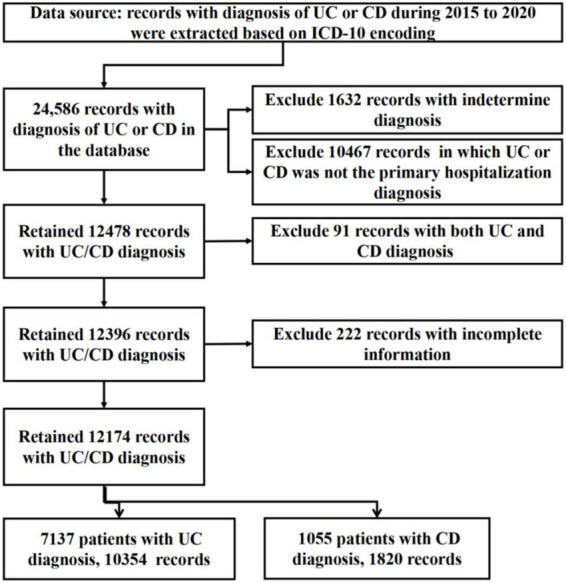
Flow chart summarizing the participants’ selection process.

The records of IBD in the database from January 1, 2015 to December 31, 2020 were retrieved based on ICD-10 coding. Extract continuous anonymous hospital records with ICD-10 K51 (UC) and ICD-10 K50 (CD) diagnostic codes. The records were independently and manually reviewed by three gastroenterologists to screen eligible patients for inclusion. Excluded records with incomplete diagnostic information to confirm a diagnosis, and records that were subsequently revised to a non-IBD diagnosis (such as intestinal tuberculosis, intestinal lymphoma, or Behcet’s disease) in the database. A flowchart summary of the data extraction process was summarized in Figure 1.

### Variables and definitions

Variables were extracted and classified, including gender, age, residence, length of stay, costs, insurance, diagnoses, surgery, and complications. Treatment options and endoscopic score were unknowable due to limited database information. For repeat admissions, the first admission was used as the demographic features. If the diagnosis of UC and CD transformed over time, the last discharge was used as the diagnostic label. According to the cost details, we identified the costs of imaging, laboratory tests, drugs, and surgery. Participants were then divided according to the interquartile range of the hospitalization costs. Records with the total hospitalization costs in the upper quartile were defined as the high hospitalization costs. Comorbidities were classified according to organ systems, and the extraintestinal manifestations of IBD were not listed separately. As gastrointestinal diseases are easily confused with the pathological manifestations of IBD, only the extra-gastrointestinal comorbidities were analyzed to avoid overdiagnosis.

### Statistical analysis

Descriptive statistics were used to report the characteristics of the patients. Continuous variables were described as the mean ± SD or median and interquartile range (IQR). The population survey data were obtained from the Yunnan Statistical Yearbook generated by the Bureau of Statistics of Yunnan, China. The relevant data can be accessed at the website: http://stats.yn.gov.cn//.

Hospitalization costs were rounded to two decimal places. Frequency tables with counts and percentages were presented for categorical variables. Spearman correlation coefficient and linear by linear association of Mantel-Haenszel chi-square tests were used to explore and demonstrate tendencies from 2015 to 2020. The chi-square test was used to detect the categorical variables. Continuous variables were analysed with Student’s t-test or the Mann-Whitney U test. Univariate and multivariate logistic regression models were used to explore factors associated with high hospitalization costs, and to calculate odds ratios (OR) and 95% confidence intervals (95% CI). The multicollinearity diagnosis was used to evaluate whether there were collinearity between variables. SPSS version 23 was used for the statistical analyses. Statistical significance was set at a *P*-value of < 0.05.

## Results

### Patient characteristics

**FIGURE 2:**
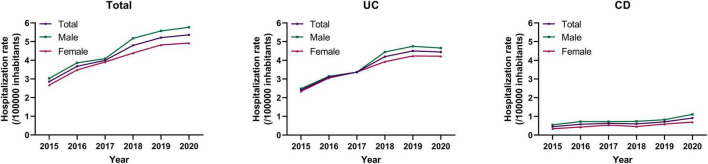
The trends of annual hospitalization rates of patients with IBD by gender in Yunnan from 2015 to 2020.

A total of 24,586 records of UC and CD were identified from the database. After screening, 12,099 records were excluded as UC or CD was not the definitive or principal hospitalization diagnosis. Ninety-one duplicate records were excluded, and 222 records were excluded due to incomplete information. We ultimately included 12,174 records [UC 10,354 (85.1%), CD 1,820 (14.9%)] from 8,192 patients for analysis (Figure 1). Males were more prevalent in both UC (1.16:1) and CD (1.64:1). Based on the population data of the Yunnan Statistical Yearbook, the hospitalization rate was analyzed from 2015 to 2020 (Figure 2), and there was no significant difference in the average hospitalization rate between male (4.58/100,000) and female (4.03/100,000). The population ranged in age from 2 to 98 years old, and patients with UC was hospitalized at an older age than those with CD [51.66 ± 15.66 versus 44.09 ± 17.5, *P* < 0.001)]. The peak age of admission were 40–59 years for patients with UC and 30–49 years for patients with CD. The patient characteristics were presented in Table 1.

**TABLE 1:** Demographic characteristics of patients.

		Total (IBD)	UC	CD	*P* value*
		*n* = 8,192	*n* = 7,137	*n* = 1,055	
Gender, *n* (%)					<0.001
	male	4,486 (54.8)	3,831 (53.7)	655 (62.1)	
	female	3,706 (45.2)	3,306 (46.3)	400 (37.9)	
Age, (years)		50.69 ± 16.11	51.66 ± 15.66	44.09 ± 17.5	<0.001
**Age group, *n* (%)**					
	0–9	52 (0.6)	41 (0.6)	11 (1)	
	10–19	254 (3.1)	178 (2.5)	76 (7.2)	
	20–29	580 (7.1)	415 (5.8)	165 (15.6)	
	30–39	1,071 (13.1)	880 (12.3)	191 (18.1)	
	40–49	1,732 (21.1)	1,536 (21.5)	196 (18.6)	
	50–59	1,931 (23.6)	1,743 (24.4)	188 (17.8)	
	60–69	1,546 (18.9)	1,412 (19.8)	134 (12.7)	
	70–79	836 (10.2)	755 (10.6)	81 (7.7)	
	≥ 80	190 (2.3)	177 (2.5)	13 (1.2)	

CD, Crohn’s disease; UC, ulcerative colitis. Demographic characteristics were extracted from the first hospital discharge records of patients during the study period. The data are presented as *n* (%) or mean ± SD.*Student’s *t*-test (continuous variables) and chi-square test (categorical variables) were used to test the differences in characteristics between UC and CD. Due to rounding, counts may not add up to the total, and percentages may not sum to 100%. *P* < 0.05 indicates statistical significance.

### The hospitalization burden of IBD

**FIGURE 3:**
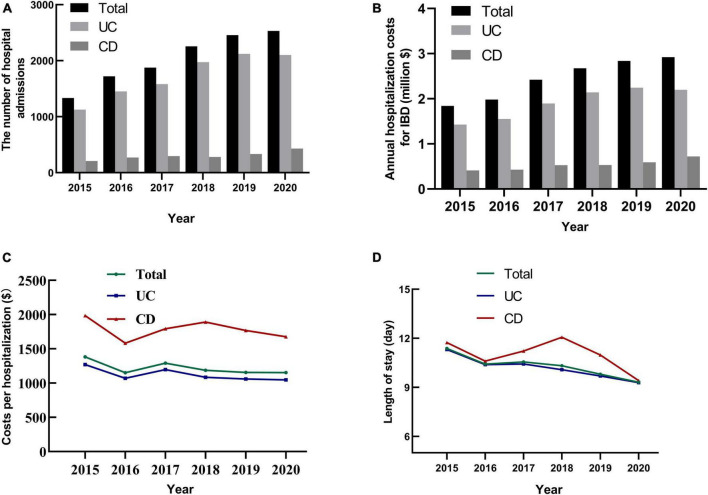
The temporal trends of hospital admissions, hospitalization costs and length of stay for IBD in Yunnan Province, China from 2015 to 2020. **(A)** The number of hospital admissions for IBD in Yunnan province increased from 2015 to 2020. **(B)** The annual hospitalization costs for IBD increased by year from 2015 to 2020. **(C)** The cost per hospitalization for UC and CD from 2015 to 2020. **(D)** The average length of stay for UC and CD decreased from 2015 to 2020.

The number of hospital admissions for IBD nearly doubled from 1,333 in 2015 to 2,531 in 2020 Figure 3A. The costs per hospitalization for CD was higher than that of UC (*P* < 0.001). The regional hospitalization burden of IBD increased from 2015 to 2020 Figure 3B, but it represented a decline in cost per hospitalization (*r* = −0.024, *P* = 0.008) Figure 3C and the length of stay (*r* = −0.098, *P* < 0.001, Figure 3D). In the subgroup analyses, the hospitalization costs for patients with UC (*r* = −0.038, *P* < 0.001) revealed a downward trend, while those for patients with CD (*r* = 0.035, *P* = 0.138) changed little over the period.

Overall, costs due to surgical intervention, laboratory tests, imaging increased from 2015 to 2020 (all *P* < 0.001). Among the identifiable cost items during the period, drug costs accounted for the highest proportion, accounting for 33% and 37.30% in patients with UC and CD, respectively (*P* < 0.001). Significantly, the percentage of drug costs decreased from 2015 to 2019, but rebounded in 2020 (Table 2). Owing to the limited information in the database, pharmacy information was not available.

**TABLE 2:** Hospitalization costs of patients with IBD in Yunnan Province from 2015 to 2020.

Variables year		2015	2016	2017	2018	2019	2020	Total
**Total hospitalization costs of IBD regional level (million $)**								
Total		1.8418356	1.98052303	2.42311039	2.67467208	2.83687737	2.92006028	14.67707875
UC		1.42895156	1.55149309	1.89455027	2.14136376	2.2465087	2.19962764	11.46249502
CD		0.41288404	0.42902994	0.52856012	0.53330832	0.59036867	0.72043264	3.21458372
**Hospitalization costs per patients**								
**UC**	Total cost per patients ($)	960.18 (617.43–1554.67)	841.34 (521.74–1309.40)	869.35 (567.60–1359.96)	841.34 (539.00–1304.83)	778.84 (515.19–1198.81)	779.97 (527.42–1190.95)	827.49 (540.11–1295.50)
	Drugs, percentage	39.20%	37.30%	35.60%	32.20%	27.80%	30.30%	33%
	Laboratory test, percentage	12.80%	14.60%	14.80%	16.70%	17.90%	19.60%	16.50%
	Imaging percentage(%)	5.80%	6.5%%	7.3%%	9%%	9.10%	9.40%	8.10%
	Surgical costs	1.50%	1.70%	1.90%	2.30%	3.00%	3.10%	2.40%
**CD**	Total cost per patients ($)	1171.71 (634.81–2033.67)	993.04 (600.82–1831.74)	1096.60 (640.86–1847.46)	1143.70 (636.23–2021.03)	1053.79 (653.04–1910.58)	999.20 (730.72–1748.17)	1057.03 (644.26–1888.78)
	Drugs	41.70%	39.20%	38.90%	36.50%	29.20%	40.20%	37.30%
	Laboratory test, percentage	11.60%	13.20%	15%	15.70%	17.40%	16.10%	15.20%
	Imaging percentage	5.60%	7.60%	8%	9%	8.80%	8.20%	8%
	Surgical costs	2.90%	3.40%	3.60%	3.20%	4.80%	4.00%	3.70%
**The length of stay in hospital per patient (day)**								
UC		10 (7–14)	9 (7–13)	9 (7–13)	9 (6–12)	9 (6–12)	8 (6–11)	9 (6–12)
CD		9 (6–16)	9 (6–14)	9 (5–14)	10 (6–14)	9 (6–14)	8 (3–13)	9 (6–14)
**Type of insurance, *n* (%)**								
	National insurance	1057 (79.3)	1403 (81.5)	1516 (80.8)	1815 (80.5)	2020 (82.2)	2149 (84.9)	9960 (81.8)
	Other forms of payment	276 (20.7)	318 (18.5)	361 (19.2)	441 (19.5)	436 (17.8)	382 (15.1)	2214 (18.2)

**FIGURE 4:**
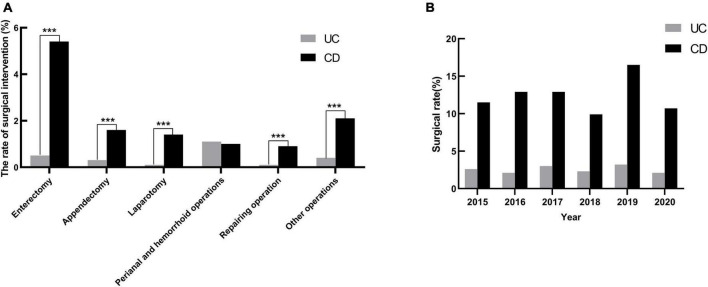
The surgical rates in hospitalized patients with IBD in Yunnan province, China, from 2015 to 2020. **(A)** In addition to perianal and hemorrhoid operations, the rate of intestinal resection, diagnostic laparotomy and repairing operation in patients with CD were higher than those in patients with UC. ***P < 0.001. **(B)** The temporal trends of surgical rates in hospitalized patients with UC and CD.

The overall surgery rate for hospitalized patients with UC was 2.5% and 12.4% for patients with CD Figure 4A. Compared with CD, patients with UC had a higher rate of endoscopy, but a lower rate of surgery. Patients with UC had a high proportion of perianal surgery, while intestinal resection accounted for the highest proportion in patients with CD. During the study period, surgery rates for hospitalized patients with CD did not decrease (*r* = −0.002, *P* = 0.932), and even increased slightly for patients with UC (*r* = 0.03, *P* = 0.002) Figure 4B.

### Complications and comorbidities

**FIGURE 5:**
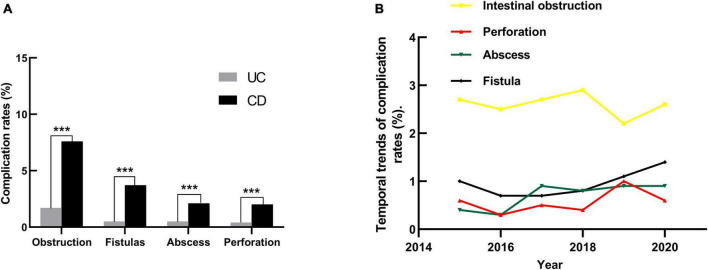
The complications rate in hospitalized patients with IBD in Yunnan province, China, from 2015 to 2020. **(A)** The complications rate in patients with UC and CD; ***P < 0.001. **(B)** The rates of intestinal obstruction, perforation, fistula and abscess in patients with IBD in Yunnan Province from 2015 to 2020. Spearman correlation coefficient tests: Intestinal obstruction (*r* = −0.003, *P* = 0.708), Perforation (*r* = 0.015, *P* = 0.093), Abscess (*r* = 0.022, *P* = 0.017), Fistula (*r* = 0.022, *P* = 0. 017). Linear by linear association of Mantel-Haenszel chi-square tests results:Intestinal obstruction (*X*^2^ = 0.107, *P* = 0.744), Perforation (*X*^2^ = 2.117, *P* = 0.146), Abscess (*X*^2^ = 6.046, *P* = 0.014), Fistula (*X*^2^ = 4.897, *P* = 0.027).

Intestinal obstruction and perforation accounted for 2.6% and 0.6% of the patients. The rate of gastrointestinal fistula and abscess were 1.0% and 0.7%, respectively. Overall, hospitalized patients with CD have a higher risk of these conditions than those with UC (*P*<0.001, Figure 5A). Trend analysis revealed no significant differences in the proportion of intestinal obstruction and perforation over the study period, while the proportion of abscesses and fistulas increased Figure 5B.

Of the included records, 8,772 (72.1%) records had comorbidities. Even excluding the duplicate hospitalization records, the proportion remained striking (70.4%). Based on the organ systems involved, the most prevalent comorbidities in the patients were endocrine and metabolic diseases (21.6%), followed by cardiovascular diseases (15.2%), hepatobiliary diseases (14.9%), respiratory diseases (12.4%), hematological disorder (10.6%), etc. A total of 189 records documented psychiatric illness, and anxiety, depression, and insomnia were the common psychiatric disorders in these patients. Infections occur frequently in hospitalized patients with IBD (18%). In particular, appendicitis was more common in patients with CD (1.5%) than in patients with UC (0.3%) (*P* < 0.001). Patients with comorbidities had longer hospital stays in both UC [(9 (6) vs. 8 (5), *P*<0.001)] and CD [9 (8) vs. 8 (9), *P*<0.001)]. Likewise, patients with comorbidities had higher costs (*P*<0.001).

### Factors associated with high hospitalization costs in patients with IBD

Records with hospitalization costs above the upper quartile were defined as having high hospitalization costs. The records were divided into binary variables according to whether the record ranked in the top 25%. Univariate and multivariate logistic regression were used to analyze the influencing factors of hospitalization costs. Univariate analysis suggested that in addition to gender, age, length of stay, and insurance, disease subtypes, complications, endoscopy, comorbidities and surgery were related to the hospitalization costs. Multivariable analysis revealed that after adjusting the factors affecting hospitalization costs such as age, length of stay and type of medical insurance, complications, comorbidities, endoscopy and surgery were still the risk factors for high hospitalization costs in patients with IBD. Surgery had a significant effect on the hospitalization costs [OR 4.87 (3.75–6.31), *P* < 0.001], while less noticed comorbidities also led to higher hospitalization costs [OR 1.72 (1.52–1.94), *P* < 0.001, Table 3].

**TABLE 3:** Factors associated with high hospitalization cost in patients with IBD using logistic regression^a^.

Variable	N	Percentage	Univariable analysis		Multivariable analysis^e^	
			OR (95% CI)	*P*	OR (95% CI)	*P*
Gender (female)^b^	5,488	45.10%	0.96 (0.89–1.05)	0.36		
Age	12,174	100%	1.01 (1–1.01)	<0.001	1.01 (1.0–1.01)	0.003
Length of stay	12,174	100%	1.27 (1.26–1.28)	<0.001	1.26 (1.25–1.28)	<0.001
Insurance status (national insurance)^c^	9,960	81.80%	0.75 (0.67–0.83)	<0.001	0.60 (0.53 –0.68)	<0.001
Disease subtypes (Crohn’s disease)^d^	1,820	14.90%	2.02 (1.82–2.24)	<0.001	2.04 (1.78–2.34)	<0.001
Complication	1,361	11.20%	1.95 (1.73–2.19)	<0.001	1.53 (1.32–1.78)	<0.001
Endoscopy	4,121	33.90%	1.59 (1.46–1.73)	<0.001	2.06 (1.86–2.28)	<0.001
Comorbidities	8,772	72.10%	1.7 (1.54–1.87)	<0.001	1.72 (1.52–1.94)	<0.001
Surgical intervention	489	4%	9.68 (7.87–11.91)	<0.001	4.87 (3.75–6.31)	<0.001

CD, Crohn’s disease; UC, ulcerative colitis; CI, confidence interval; OR, odds ratio; 95% confidence interval data were rounded to keep two decimal places. ^a^Whether records belonged to the high-cost group were classified as a binary variable and included as the dependent variable in the logistic regression analysis. ^b^Female versus male. ^c^National insurance versus other forms of payment. ^d^Chronic diseases versus ulcerative Colitis. ^e^Regression model included age, length of stay, insurance status, disease subtypes, complication, endoscopy, comorbidities and surgical intervention.

## Discussion

Paradigm shifts in modern therapeutics and management strategies have largely shifted the management of moderate-to-severe IBD from the hospital to the outpatient clinic. Hospitalization rates for a primary diagnosis of IBD were stable in the Western world. While, newly industrialized countries have rapidly increasing hospitalization rates, where incidence is rapidly increasing and access to advanced medical therapies is different (17). Overall, the escalating prevalence of IBD and an aging population have contributed to an increasing hospitalization burden and an increasing burden on global health care systems. Recent studies have suggested that the increasing prevalence of IBD and the use of biologic drugs in recent years have increased the cost of IBD (18). Notably, there appears to be a lack of robust data on the prevalence and hospitalization burden of IBD in China. Based on a provincial database in China, the study assessed the real-world hospitalization burden of IBD in an underdeveloped area with low incidence. The Gross Domestic Product of Yunnan Province increased from 1.496 billion (yuan) in 2015 to 2.45219 billion (yuan) in 2020. The increased hospital admissions of IBD corresponds to the increase in incidence and prevalence of the region. Even in the low-incidence areas such as Yunnan Province, the increasing hospitalization burden of IBD is becoming a problem.

IBD peaks at the most productive age, and direct health costs associated with disease-related morbidity and the indirect costs associated with lost productivity and reduced quality of life are considerable (6). Hospitalization is one of the major health care costs drivers for IBD (3). The widespread use of biologics increased the cost of treatment in patients with IBD (5, 19, 20). From 2015, biologics were introduced to an increasing number of patients in Yunnan Province. The cost per hospitalization and drug costs rate for patients with IBD fluctuated during the study period, but were lower than in Western countries (13). A possible reason is that the patients cannot afford biologics in Yunnan, and the utilization rate was lower than that in developed areas. In 2020, we observed an increase in the drug costs ratio in Yunnan Province, and the medical insurance expands coverage to infliximab in the same year. Our data suggested that the cost per hospitalization for the patients did not increase during the period. Even so, the costs was still a burden for most patients in Yunnan Province (per capita GDP was ¥57,686).

Complications can be fatal for patients with IBD, and reducing or preventing them is a major goal in therapy. Surgery rates and complication rates for patients with IBD have decreased in some areas, which was a testament to the advances in therapy (21, 22). The complication rate of hospitalized patients with IBD in Yunnan Province did not decrease during the study period. In addition to bringing discomfort to the patients, complications are also one of the factors that lead to increased hospitalization costs. Surgery may be an option for IBD due to complications or certain difficulties. In our study, patients with CD or UC had a higher rate of surgery than another study from a developed city in China (13). Trend analysis revealed a slight increase in the rate of surgery among hospitalized patients with UC. This may be due to an increase of severe cases as the prevalence of IBD increases. The high rates of surgery and complications were concerning, and suggested the need to optimize treatment strategies for IBD in this region.

Earlier historical and epidemiological studies from the Western world reported a higher incidence of UC. Owing to rapid changes to environmental factors, gene susceptibility and dietary habits, the prevalence of CD is now higher than that of UC in many Western nations (23). Patients with UC accounted for 85.1% of our study population, which might be due to the relatively low incidence of CD in Yunnan Province (9). We have observed a sustained growing of hospital admissions for CD, which may coincide with the fact that CD is catching in China (12). In addition, patients with CD had higher rates of complications and surgery in the study. This may be due to the complex disease behavior, difficulties in differential diagnosis, and the high demand for surgery. The cost per hospitalization of CD was higher than that of UC and did not decrease over the study period. Since CD has more severe complications and consumes more resources, effective countermeasures are needed to counter the likely steady increased prevalence of CD, given the western experience.

Comorbidities defined as a group of diseases associated with a given condition of IBD (7, 24). Comorbidities in patients with IBD were associated with delayed diagnosis, medication safety, readmission, adverse events, mortality and treatment cessation (25–29). Comorbidities also prolong hospital stays and lead to increased hospital costs (28, 30–32). Patients with IBD were associated with increased psychiatric comorbidities compared with the general population, and which were associated with excess healthcare utilization (33). The presence of rheumatism, acid-related diseases and pain more than doubled the total cost for IBD patients (34). The composition of comorbidities varied across regions (28, 34–36). To avoid overdiagnosis, we only delineated the pattern of the extra-gastrointestinal comorbidities of IBD in our study. We found that comorbidities were common in patients with IBD and were associated with high hospitalization costs. According to the affected organ system involved in comorbidities, the top three were endocrine and metabolic diseases, cardiovascular diseases and hepatobiliary diseases. Mesonero et al. also reported a high rates of metabolic and cardiovascular diseases in patients with IBD, but followed by mental-behavioral disorders (36). The incidence of psychological disorders in IBD patients has been demonstrated in studies (33, 37). Psychological disorders had a low prevalence in our study, which may reflect the insufficient attention. Notably, the prevalence of anemia in our study was 9%, which was lower than in other studies (38). We observed fewer diagnoses of nutritional deficiencies, including vitamin B12, vitamin D, and iron deficiencies. This may be due to the inadequate awareness of these problems that frequently exist in patients with IBD. Studies on other immune-mediated diseases have demonstrated that the management of comorbidities could be cost-effective (39, 40), but limited studies and targeted guidelines on comorbidities of IBD were available.

In addition to surgery and comorbidities, the type of insurance, endoscopy, and age were associated with hospitalization costs. As the population ages, the increasing comorbidities and multidrug interactions will be a challenge for disease management in the near future. Of note, the influence of national insurance on hospitalization cost confirms the guiding role of government in disease prevention and control. Endoscopy plays an important role in the monitoring of IBD. In view of this, exploring the reasonable frequency of endoscopy may be a cost-effective method that can both take into account disease management.

As with any retrospective study, our study has some limitations. Due to database access restrictions, it was difficult to track the exact time of diagnosis and drugs informations, nor to explore the impact of disease activity. Consistent with other studies based on hospital databases, diagnosis based on ICD-10 coding has the potential for overestimation and misclassification. Nevertheless, the increased burden of IBD is evident in Yunnan, an area of low prevalence and emerging industrialization. The high surgical rates, complications, and comorbidities of patients with IBD in newly industrialized regions should be of concern. After all, they can complicate disease management and lead to higher hospitalization costs in these areas. Reducing complications and the need for surgery by promoting behavioral change, managing comorbidity, may be effective means of controlling the increasing burden of IBD.

## Data availability statement

The raw data supporting the conclusions of this article will be made available by the authors, without undue reservation.

## Ethics statement

The studies involving humans were approved by the Ethics Committee of the First Affiliated Hospital of Kunming Medical University approved the study (IRB No. 2022-L-90). The studies were conducted in accordance with the local legislation and institutional requirements. Written informed consent for participation was not required from the participants or the participants’ legal guardians/next of kin in accordance with the national legislation and institutional requirements.

## Author contributions

YT: Writing–original draft, Investigation. ML: Formal analysis, Writing–original draft. HG: Data curation, Writing–review and editing. JW: Writing–review and editing, Investigation. YS: Data curation, Writing–review and editing. FZ: Writing–review and editing, Investigation. HL: Writing–review and editing, Investigation. LH: Formal analysis, Writing–review and editing. MG: Writing–review and editing, Investigation. JN: Writing–review and editing, Conceptualization. YM: Writing–review and editing, Funding acquisition.
